# Efficacy and safety of perioperative magnesium on postoperative pain in adult patients undergoing cardiothoracic surgeries- a systematic review and meta-analysis

**DOI:** 10.1186/s13019-026-04252-0

**Published:** 2026-05-07

**Authors:** R. K. Swetha, Balasubramaniam Gayathri, Madhumitha Haridoss, Deepakraj Kuppuraman, Sananthya karthikeyan

**Affiliations:** 1 Department of Anesthesiology, SRM Medical College Hospital and Research Centre, Faculty of Medicine and Health Sciences, SRM Institute of Science and Technology, Kattankulathur, Chengalpattu, Tamil Nadu India; 2 Division of Medical Research, SRM Medical College Hospital and Research Centre, Faculty of Medicine and Health Sciences, SRM Institute of Science and Technology, Kattankulathur, Chengalpattu, Tamil Nadu India

**Keywords:** Magnesium, Postoperative pain, Thoracic surgery, Cardiac surgery, Opioid analgesics

## Abstract

**Background:**

Cardiothoracic surgeries such as CABG, valve replacement, and thoracotomy are associated with significant postoperative pain, for which opioids remain the primary treatment despite notable adverse effects and tolerance. Magnesium, an NMDA receptor antagonist with analgesic and cardioprotective properties, has been suggested as a perioperative adjuvant to improve pain control. This systematic review and meta-analysis examined the efficacy and safety of perioperative magnesium for postoperative pain relief in adult cardiothoracic surgery patients.

**Methods:**

The review followed PRISMA guidelines and was registered with PROSPERO (CRD420251049449). A search was conducted in PubMed, EMBASE, Scopus, and CENTRAL up to 14 May 2025 for randomized controlled trials comparing perioperative magnesium with placebo or other analgesics in adult patients undergoing cardiothoracic surgery. Pain scores, opioid consumption (MME), time to rescue analgesia, length of hospital stay, and adverse events were analyzed. Random-effects meta-analyses were performed using R Studio (v4.3.2) with mean difference (MD) or risk ratio (RR) and 95% confidence intervals (CI).

**Results:**

Ten RCTs involving 1,140 participants were included. Magnesium significantly reduced pain intensity compared with placebo at 24 h postoperatively (MD − 0.85, 95% CI: −1.53 to − 0.16), although the effect did not reach the minimal clinically important difference. No significant differences were observed when magnesium was compared with active analgesics. For all other outcomes, including opioid consumption (MME), time to first rescue analgesia, and length of hospital stay, no significant differences were found in pooled analyses versus either placebo or other analgesics. Heterogeneity was substantial across most outcomes (I² > 75%), and no major adverse events were reported.

**Conclusion:**

Perioperative magnesium shows small and safe analgesic effects after cardiothoracic surgery, but the clinical relevance remains uncertain due to substantial heterogeneity and variable dosing. Further robust trials are needed to define its efficacy and optimal use.

**Supplementary Information:**

The online version contains supplementary material available at 10.1186/s13019-026-04252-0.

## Introduction

Cardiothoracic procedures like coronary artery bypass grafting, heart valve replacement, or mixed procedures often lead to excessive pain and discomfort post-surgery. Managing the post-operative pain is crucial; inadequate analgesia might weaken respiratory function, delay mobilization, and prolong stay in intensive care units [1]. Traditionally, opioids have formed the foundation of postoperative analgesia in cardiothoracic patients. However, their use is often limited by adverse effects, including respiratory depression, nausea, vomiting, constipation, sedation, and the potential for tolerance and dependence [2]. These limitations underscore the need for the safest multimodal analgesic plan of action that optimizes pain relief while minimizing opioid consumption and its related complications.

In recent years, magnesium has received increasing attention in perioperative pain care owing to its unique pharmacological profile. Magnesium acts as an NMDA receptor antagonist, modulating central sensitization and inhibiting the excitatory neurotransmission mechanism that leads to postoperative pain in surgical patients [3]. Additionally, magnesium plays a key role in muscle relaxation, vasodilation, and cardiac electrophysiology. Perioperative magnesium supplementation has been linked to cardio-protective benefits in addition to its analgesic effects, especially in reducing the incidence of postoperative atrial fibrillation and arrhythmias in patients undergoing cardiac surgery [4, 5].

Previous research on magnesium supplementation in various other surgeries has revealed possible opioid-sparing and analgesic effects [6]. However, sufficient clinical evidence for cardiothoracic surgery remains limited. While some RCTs on magnesium supplementation have reported improvement in pain scores, reduction in opioid consumption, and minimization of length of the hospital stay, some studies have reported no significant benefits [7, 8]. Additionally, variation in study designs, dosage regimens, timing of administration, and outcome measures complicates interpretation and limits generalizability.

Given the current ambiguity in the literature, a comprehensive systematic review of available clinical evidence is warranted. Although magnesium has been evaluated in meta-analyses across various surgical populations, its specific effectiveness in cardiothoracic surgery remains unclear [9, 10]. Addressing this gap is clinically important, as optimizing perioperative analgesia in this high-risk population may help reduce complications, enhance recovery, and facilitate the implementation of multimodal pain management strategies.

Therefore, this systematic review and meta-analysis aim to evaluate the efficacy and safety of perioperative magnesium administration in reducing postoperative pain among adult patients undergoing cardiothoracic surgery. Additionally, it will assess its impact on opioid consumption, length of hospital stays, and other relevant postoperative outcomes. By synthesizing current evidence, this review seeks to clarify the role of magnesium in perioperative pain management and inform future clinical practice.

## Methods

This systematic review and meta-analysis was conducted in accordance with the Preferred Reporting Items for Systematic Reviews and Meta-Analyses (PRISMA 2020 ) guidelines, and the protocol was registered with PROSPERO (CRD420251049449).All deviations from the protocol were made a priori and before data extraction or analysis to ensure transparency and minimize bias. The list of deviations, along with reasoning, is presented in Supplementary Table 1.

### Information sources

A systematic search was conducted from inception to 14 May 2025 in PubMed, EMBASE, Scopus, and the Cochrane Central Register of Controlled Trials (CENTRAL) to identify relevant studies. The search strategy was developed and verified by the PRESS checklist [11]. The reference lists of included studies were also screened to identify any additional eligible records. Full database-specific strategies, including Mesh/Emtree terms, are provided in Supplementary Table 2.

### Inclusion and exclusion criteria

Studies were eligible for inclusion if they were randomized controlled trials that involved adult patients (aged 18 years or older) undergoing cardiothoracic surgery, valve repair or replacement surgery, thoracic aortic surgery, or lung resections (either thoracotomy or video-assisted thoracoscopic surgery). The study intervention involved administering magnesium through any route, specifically during the perioperative phase, for the management of postoperative pain before, during, or after surgery. The comparator was a placebo for the use of standard care or an analgesic regimen that did not utilize magnesium. Studies were required to include at least one of the following outcome measures in order to be included in the review: pain scores measured after surgery using the Visual Analogue Scale (VAS), opioid consumption (MME), rescue analgesic requirement, or adverse events such as nausea, vomiting, arrhythmias, or respiratory depression.

Studies were excluded if they were conducted in pediatric patients (< 18 years), patients undergoing non-cardiothoracic surgery, or where magnesium was administered for non-analgesic purposes (e.g., arrhythmia prophylaxis, seizure disorder, or electrolyte correction). We also excluded trials where magnesium was given in conjunction with multiple adjuvants, and it was not possible to isolate magnesium as the analgesic component. Non-randomized studies, animal studies, abstracts, case reports, reviews, letters, editorials, and expert opinions were not considered.

### Study selection and data extraction

Two reviewers (RK and DK) independently screened titles and abstracts per inclusion criteria, then reviewed full texts for further selection. The disagreement was resolved in consultation with the third reviewer (HM). A data extraction form was created in MS Excel 365 and standardized by pilot data extraction for three studies. Data were extracted from the included trials by reviewer 1 (RK) and verified for accuracy and consistency by reviewer 2 (DK). Opioid doses (including morphine and fentanyl) were converted to morphine milligram equivalents (MME) using standard conversion factors prior to pooling to ensure comparability across studies [12].The extracted information covered bibliographic details (study ID, study label, first and corresponding authors with contact information, journal, year of publication, and country); study characteristics (design, duration, population, type of surgery, and sample size); and group allocation (intervention, comparator 1, and comparator 2) along with demographic data such as age and sex distribution across groups. Intervention-related variables included route of administration, dosing regimen, frequency, and any specific remarks. Outcome domains comprised postoperative pain scores measured using the VAS at 24 and 48 h with mean and standard deviation; analgesic requirements (mean dose and variability across groups); time to first analgesic requirement; hemodynamic parameters (blood pressure and heart rate); length of hospital stay; and adverse events, including arrhythmias and bradycardia. For each outcome, both absolute values and comparative analyses between the intervention and comparator arms were documented. Detailed intervention characteristics, including route of administration, timing, and dosing regimens of magnesium for each included study, are provided in the study characteristics table (Supplementary Table 5).

### Risk of bias assessment

We evaluated the risk of bias using the Cochrane Risk of Bias tool 2 (RoB-2) at the study level [13]. The risk of bias of the included randomized controlled trials was assessed independently by two reviewers (RK and DK). This tool enables a systematic and comprehensive evaluation of potential sources of bias in randomized trials included in systematic reviews and meta-analyses. Each study was assessed for and assigned a low, unclear, or high risk of bias across the following domains: bias arising from the randomization process, deviations from intended interventions, missing outcome data, measurement of outcomes, and selection of the reported result. Any disagreements in risk of bias assessments were settled through discussion and consensus with the third reviewer (HM).

Although the protocol outlined the primary outcomes, certain methodological refinements were made a priori before formal data analysis to enhance consistency and interpretability. Initially, specific postoperative time points for outcome assessment were not predefined in the protocol. However, during preliminary data extraction, it was observed that included studies reported pain outcomes at multiple time intervals (e.g., 4, 6, 12, 24, and 48 h). Among these, 24 h was the most frequently reported time point across the majority of studies. Therefore, postoperative pain at 24 h was designated as the primary outcome, while pain at 48 h was considered a key secondary outcome, to ensure comparability across studies.

In addition, length of hospital stay (LOS), although not prespecified in the protocol, was incorporated as a secondary outcome, as several included studies reported this parameter, and it was considered clinically relevant for assessing the overall effectiveness of perioperative magnesium.

The review was restricted to adult populations (≥ 18 years), as defined in the protocol. Furthermore, although the protocol initially specified the use of a different statistical platform, the analysis was conducted using R software (version 4.3.2), which provided enhanced flexibility and reproducibility, particularly for generating forest plots and conducting advanced meta-analytic procedures. All these decisions were made prior to formal analysis and are reported to maintain transparency and methodological rigor.

### Data synthesis

Meta-analyses were performed in R Studio (v4.3.2) using the Metafor package (v3.8-2) with REML random-effects models. Given the anticipated clinical heterogeneity across studies, including differences in routes of magnesium administration, dosing regimens, and comparator groups, a random-effects model was considered appropriate to account for between-study variability. Given this heterogeneity, the meta-analysis was conducted with an exploratory objective, and the findings should be interpreted cautiously rather than as a single definitive pooled estimate. Pain was reported in 0–10 VAS, where higher indicated greater pain. The mean difference (MD) in pain score between magnesium (Mg) and the comparator was pooled in a meta-analysis, where a negative MD favors magnesium. Although not specified in the protocol, a between-group reduction of ≥ 1 point on the 0–10 VAS was considered the Minimal Clinically Important Difference (MCID), based on evidence from previous literature, and was applied during the interpretation of the pooled estimates [16]. Other continuous outcomes (rescue analgesia required, time to analgesia, and length of stay) were pooled as MD with 95% CI and dichotomous outcomes (arrhythmias and bradycardia) as RR with 95% CI. Heterogeneity was assessed using I² (low: ~25%, moderate: ~50%, high: ~75%) and Chi-square tests [14, 15].Subgroup analyses were not performed due to an insufficient number of studies. However, separate pooled analyses were carried out for pain at 24 h and 48 h postoperatively. All outcomes were stratified by comparison type: magnesium vs. placebo/normal saline and magnesium vs. other analgesics. Sensitivity analyses included leave-one-out and exclusion of high-risk-of-bias studies. Publication bias assessment using funnel plots and Egger’s test was planned for outcomes with ≥ 10 studies but was not performed due to fewer contributing studies [17].

## Results

### Study selection

**Fig. 1:**
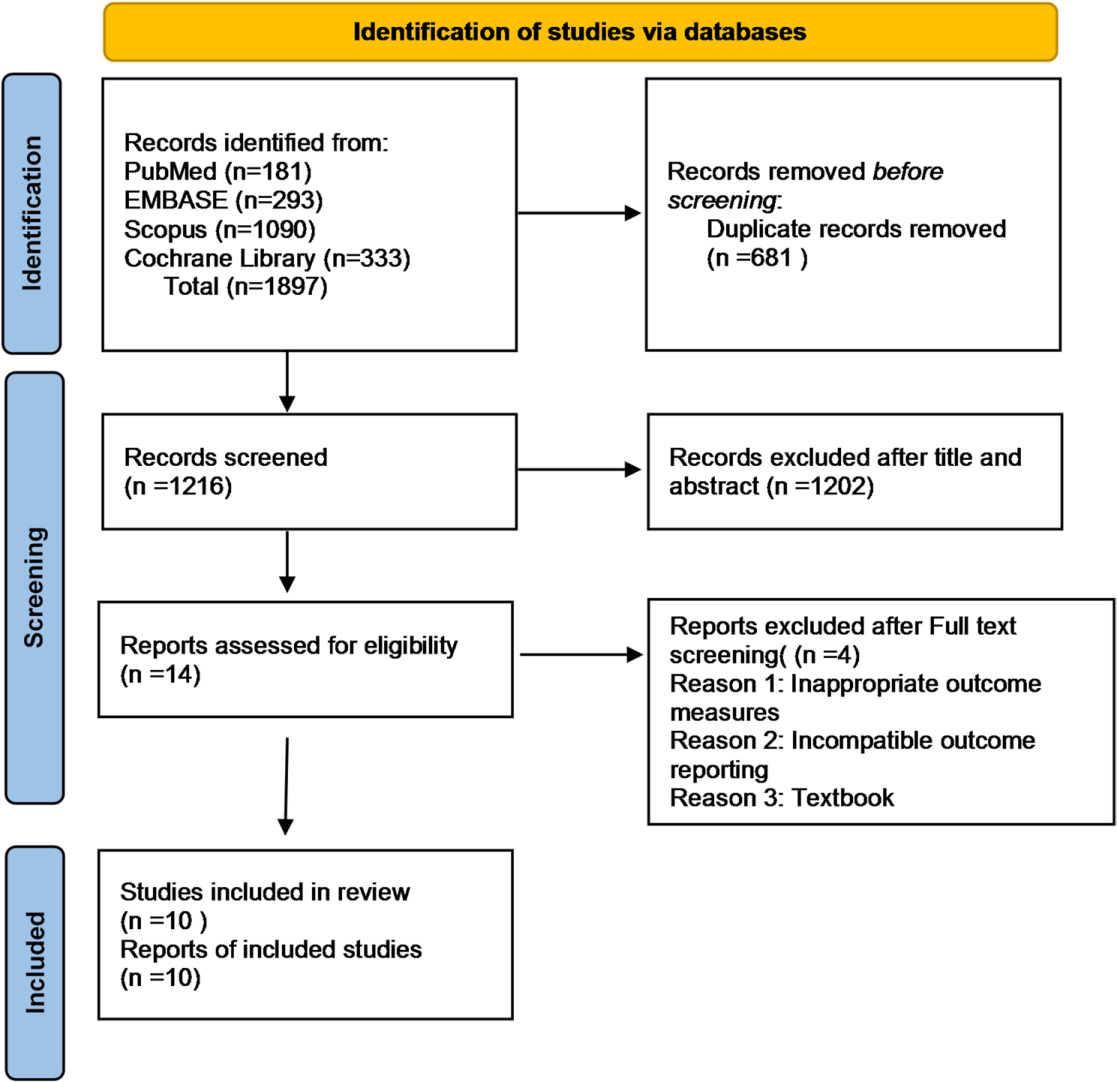
Prisma 2020 flow diagram

A systematic literature search conducted in 14 May 2025 identified a total of 1,897 records across four databases: PubMed (*n* = 181), EMBASE (*n* = 293), Scopus (*n* = 1,090), and the Cochrane Library (*n* = 333). Before screening, 681 duplicate records were removed, resulting in 1216 studies for eligibility assessment. Following initial screening, 1202 records were excluded as they clearly did not meet the inclusion criteria based on title and abstract review. The full texts of 14 included articles were then retrieved and assessed for eligibility by two independent reviewers. Of these, 4 articles were excluded after full-text assessment due to reasons including non-randomized study design, paediatric population, magnesium administered for non-analgesic purposes such as arrhythmia prophylaxis or electrolyte correction, non-cardiothoracic surgical procedures, insufficient data for meta-analysis, and studies where magnesium was combined with multiple adjuvants without isolatable analgesic effects. The list of excluded studies is presented in Supplementary Table 4. Ultimately; ten randomized controlled trials met al.l inclusion criteria and were included in the systematic review and meta-analysis [18–27]. The study selection process is illustrated in the PRISMA flow diagram (Fig. 1).

### Characteristics of Included Studies

A total of ten randomized controlled trials published between 2005 and 2018 were included in this review, evaluating the efficacy and safety of magnesium compared to placebo/analgesia in patients undergoing cardiothoracic surgery. Studies were conducted across diverse geographical regions, including Egypt, Iran, India, Turkey, Austria, Korea, and Croatia. Most studies were randomized controlled trials, either double-blind or prospective in design, involving patients undergoing major cardiothoracic or vascular surgeries such as coronary artery bypass grafting (CABG)(*n* = 3) [19, 22, 23], open-heart procedures (*n* = 1) [18], thoracotomy (*n* = 3) [20, 24, 25], video-assisted thoracic surgery (VATS)(*n* = 1) [21] Vascular(*n* = 1) [26], and Lobectomy(*n* = 1) [27]. The study population primarily consisted of adult patients scheduled for elective surgeries, with sample sizes ranging from 24 to 218 participants.

Among the included studies, nine studies assessed Magnesium sulphate, and only one study assessed Magnesium gluconate [22]. Across the studies, magnesium was administered as an adjuvant along with fentanyl or morphine, either with or without local anaesthetic agents such as bupivacaine, ropivacaine, or levobupivacaine, through various routes including epidural (*n* = 6) [20, 21, 24–27], intravenous (*n* = 3) [20, 22, 23], and local infiltration (*n* = 1) [18]. Most studies included two comparator groups: one receiving normal saline or placebo, and another receiving other analgesic agents such as non-steroidal anti-inflammatory drugs (NSAIDs) or opioids (codeine, ketorolac, remifentanil), either with or without local anaesthetic agents. Pain outcomes were commonly assessed at 4, 6, 8, 12–48 h using the visual analogue scale (VAS) Table 1.

The overall risk of bias among the included RCTs was generally low, as illustrated in Supplementary Table 3. Most studies were judged to have a low risk of bias across all five domains assessed by the Cochrane Risk of Bias 2.0 tool. Specifically, the domains of bias due to deviations from intended interventions (D2), bias due to missing outcome data (D3), and bias in selection of the reported results (D5) consistently demonstrated low risk across nearly all studies. A small number of trials were rated as having some concerns, primarily in the domains of bias arising from the randomization process (D1) and bias in measurement of the outcome (D4). These concerns were generally related to incomplete reporting of random sequence generation or allocation concealment, as well as the potential influence of unblinded outcome assessment on pain-related endpoints. Only one study [23] was assessed as having a high risk of bias in the randomization process due to insufficient methodological detail and unclear allocation concealment procedures. Importantly, no major concerns were identified regarding deviations from the intended intervention, missing data, or selective outcome reporting. Overall, the methodological quality of the included trials was satisfactory, indicating that the pooled quantitative estimates derived from these studies are based on evidence of generally reliable internal validity.

## Quantitative and qualitative synthesis

### Pain Intensity at 24 hours

#### Magnesium vs. placebo/normal saline

**Fig. 2:**
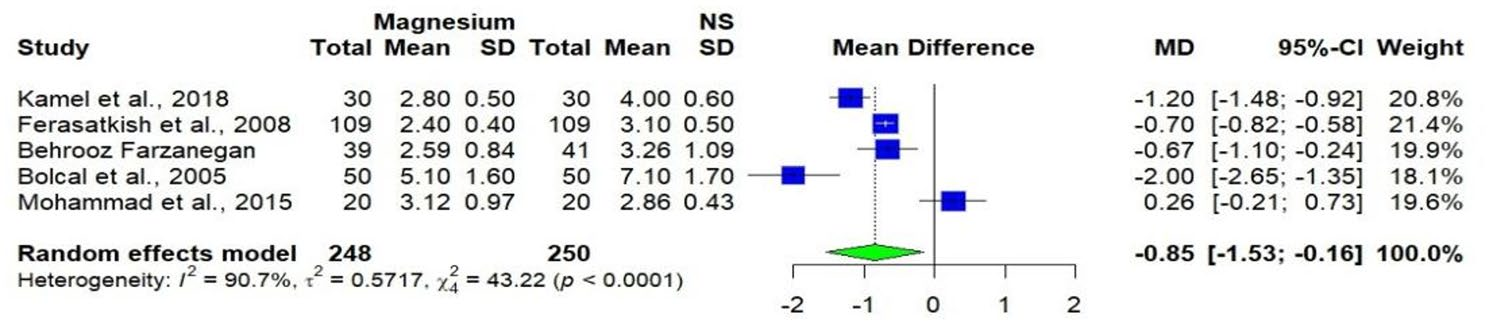
Forest plot showing pooled mean difference in pain score in 24 h between magnesium and saline groups

Among the ten included studies, five [18–20, 23, 24] compared the Mg and placebo/Normal saline groups, assessing pain at 24 h post-surgery with a pooled sample size of 248 and 250, respectively. The overall mean difference in VAS score between the groups was − 0.85 (95% CI: -1.53 to -0.16), which was statistically significant, suggesting a lower pain score in the Mg group **(**Fig. 2**).**

However, this difference did not reach the MCID of 1.0 points and, therefore, may not be clinically meaningful. The observations also varied widely across the individual studies, as indicated by substantial heterogeneity (I² = 90.7%, *p* < 0.0001). The observed heterogeneity could be attributed to differences in the type of surgery, concomitant analgesics and anesthetics, route of Mg administration, dose of Mg, and other factors. However, this could not be explored in subgroup analysis due to the insufficient number of studies. Hence, we conducted leave-one-out sensitivity analysis (excluding one study at a time), which showed a pooled mean difference ranging from − 1.09 (95% CI: -1.64 to -0.54) to -0.60 (95% CI: -1.18 to -0.02); however, the level of heterogeneity remained unchanged across sensitivity analyses. (Supplementary Fig. 1)

#### Magnesium vs. active analgesics

**Fig. 3:**
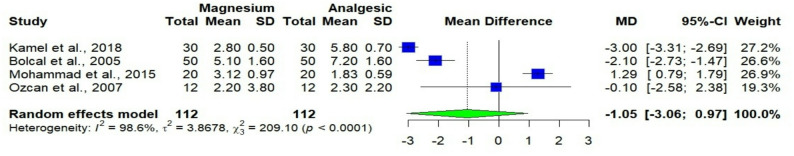
Forest plot showing pooled mean difference in pain score in 24 h between magnesium and analgesia groups

Among the four included studies [18, 23–25] comparing magnesium with analgesia, the pooled sample sizes in the magnesium and analgesia groups were 112 and 112, respectively. The overall mean difference in VAS score between the groups was − 1.05 (95% CI: -3.06 to 0.97), indicating a trend toward lower pain scores in the magnesium group, although this difference was not statistically significant (Fig. 3**)**.

Heterogeneity was observed to be high across studies (I² = 98.6%, *p* < 0.0001), possibly due to differences in the type of surgery, magnesium dosage, timing, and concomitant analgesic regimens. In leave-one-study -out sensitivity analysis, the pooled mean difference ranged from − 2.19 (95% CI: -3.39 to -0.98) when Mohammed et al., 2015 [24] was excluded to -0.32 (95% CI: -2.49 to 1.85) when Kamal et al., 2018 [18] was omitted, indicating that the overall findings were influenced by individual study effects. (Supplementary Fig. 2). Across all studies, no significant adverse effects were observed, confirming the safety of magnesium within therapeutic ranges.

#### Pain intensity at other time points

Meta-analysis was not feasible for outcomes at 4, 6, 8, 12, or 48 h due to the limited availability of comparable data. Narratively, magnesium demonstrated early analgesic benefits (2–6 h), which were often sustained up to 12 h and variably through 24–48 h, depending on the dose, route, and type of procedure.

### Route- and procedure-specific patterns

Epidural magnesium [20, 21, 24, 26, 27] was reported to be associated with reductions in pain scores at multiple postoperative time points. In contrast, studies using intravenous administration [19, 22, 25] more commonly reported reductions in opioid consumption, with less consistent effects on VAS scores.

Notably, some studies reported opioid-sparing effects in the absence of significant differences in pain scores, suggesting that magnesium may influence analgesic requirements or opioid responsiveness in addition to pain perception.

Decreased rescue analgesia use, along with lower incidences of postoperative shivering, nausea, and vomiting, was reported in several studies, suggesting potential broader recovery benefits; however, these findings were not uniformly observed.

### Opioid consumption

To further evaluate the clinical impact of magnesium on postoperative pain management, cumulative opioid consumption (MME) was compared between groups by pooling the mean difference in total opioid consumption, expressed as morphine milligram equivalents (MME) in the magnesium versus normal saline/placebo and magnesium versus active analgesic groups. Seven studies [18–20, 22–24, 26] (n = 597; magnesium = 297, control = 300) compared magnesium with a placebo, showing a pooled mean difference of − 9.69 mg MME (95% CI: −19.40 to 0.02), indicating lower analgesic use in the magnesium group; however, the difference was not statistically significant. (Supplementary Fig. 3) Four studies [18, 23–25] (n = 224; 112 per group) compared magnesium with other analgesic adjuvants, yielding a pooled mean difference of) − 36.70 mg MME (95% CI: −98.61 to 25.21), again showing a non-significant reduction in opioid requirement. (Supplementary Fig. 4) Considerable heterogeneity was observed across both analyses (I² = 98.8% and 100%, respectively), likely reflecting variations in surgical type, magnesium dosage, timing of administration, and concurrent use of analgesics. (Supplementary Fig. 5) Sensitivity analyses indicated that the summary estimates were influenced by individual studies. Leave-one-out sensitivity analysis showed that the pooled mean difference ranged from − 49.14 (95% CI: −129.62 to 31.35) to − 5.16 (95% CI: −11.90 to 1.57). Exclusion of any single study did not change the overall non-significant effect, and high heterogeneity (I²=100%) persisted across all analyses.” Substantial heterogeneity was observed, with notable variability in magnesium administration routes, dosing regimens, and comparator groups across the included studies.Nevertheless, the degree of heterogeneity did not change materially, indicating that variability among studies persisted despite the exclusion of outliers. (Supplementary Fig. 6)

### Time to first rescue analgesia

Given the observed trends in reduced opioid consumption, subsequent analysis focused on the duration of analgesic efficacy, measured as the time to first rescue analgesia. The time to first analgesic administration was evaluated in studies comparing magnesium with either a placebo or active analgesic adjuvants. Six trials [18, 20–22, 24, 26] (*n* = 395; magnesium = 195, control = 200) compared magnesium with placebo, showing a pooled mean difference of 51.85 min (95% CI: −2.83 to 106.52), indicating a trend toward a longer pain-free interval in the magnesium group, though not statistically significant. (Supplementary Fig. 7). Two additional studies [18, 24] (*n* = 100; 50 per group) compared magnesium with other analgesic agents and reported a pooled mean difference of 96.53 min (95% CI: −145.53 to 338.58), also favoring magnesium without statistical significance. (Supplementary Fig. 8) Considerable heterogeneity was observed across both analyses (I² = 97.3% and 99.5%, respectively), reflecting variations in surgical type, dosing regimens, and timing of magnesium administration. (Supplementary Fig. 9).

### Length of hospital stay

Finally, to assess whether improved pain control and recovery profiles translated into faster postoperative recovery, length of hospital stay was compared between magnesium and control groups. Length of hospital stay was reported in studies comparing magnesium with either placebo or active analgesic agents. Two trials [18, 23] (*n* = 160; 80 per group) compared magnesium with placebo and showed a pooled mean difference of − 0.42 days (95% CI: −1.40 to 0.56), indicating a slightly shorter hospital stay in the magnesium group, though the difference was not statistically significant. (Supplementary Fig. 10) Three additional studies [18, 23, 25] (*n* = 184; 92 per group) compared magnesium with other analgesic adjuvants, yielding a pooled mean difference of − 0.19 days (95% CI: −1.24 to 0.85), again showing no significant difference between groups. (Supplementary Fig. 11) Leave-one-out sensitivity analysis demonstrated that the pooled mean difference ranged from − 0.70 (95% CI: −0.98 to − 0.42) to 0.39 (95% CI: −0.19 to 0.98). Exclusion of any single study did not materially alter the overall non-significant effect, while substantial heterogeneity persisted (I² ≈ 81.7%). The pooled estimates did not materially change the overall conclusions. (Supplementary Fig. 12).

## Discussion

This systematic review and meta-analysis synthesized evidence from ten randomized controlled trials evaluating the perioperative use of magnesium for postoperative analgesia in adult patients undergoing cardiothoracic surgery. Magnesium was associated with a statistically significant reduction in pain intensity at 24 h compared with the placebo; however, the magnitude of this difference did not exceed the minimal clinically important difference and is thus unlikely to be clinically meaningful. Across other outcomes, including opioid consumption (MME), time to first rescue analgesia, and length of hospital stay, magnesium showed favorable trends but no statistically significant effects. Considerable heterogeneity was observed for all pooled outcomes, reflecting differences in surgical procedures, magnesium dosing regimens, routes of administration, and concomitant analgesic strategies. Despite this variability, magnesium demonstrated a good safety profile, with no major adverse events reported, supporting its potential role as a safe adjunct within multimodal analgesia protocols for cardiothoracic surgery.

Several factors likely contributed to the substantial heterogeneity observed across pooled analyses. Clinical heterogeneity arose from the inclusion of diverse cardiothoracic procedures (CABG, valve surgery, thoracotomy, and VATS), which differ in baseline pain burden, surgical trauma, and recovery trajectories. Methodological variability included differences in magnesium dose (low versus high, bolus versus continuous infusion); timing (preoperative, intraoperative, or postoperative initiation); and route of administration (intravenous, epidural, or wound infiltration), as well as variation in concomitant opioid and non-opioid analgesic regimens. Outcome heterogeneity further complicated comparisons, with trials using different pain scales, assessment time points, and definitions of opioid consumption (MME) or rescue analgesia, limiting the precision and interpretability of pooled estimates [28]. Sensitivity analyses showed that individual studies appreciably influenced summary effects and that high I² values persisted, indicating genuine between-study variability rather than sampling error alone​ [29]. A key limitation of this review is the considerable clinical heterogeneity among included studies, particularly in terms of magnesium administration routes and comparator groups. This variability may limit the interpretability of pooled estimates and reduces the certainty of conclusions. Although a random-effects model was applied, the pooled findings should be interpreted with caution, as they may represent an average effect across clinically diverse settings rather than a single uniform treatment effect. Furthermore, given the substantial heterogeneity across included studies, the findings of this meta-analysis should be considered exploratory in nature rather than definitive estimates of effect.Due to the limited number of included studies and substantial heterogeneity, formal subgroup analyses (e.g., by route of administration or type of surgical procedure) were not performed, and any apparent patterns should therefore be interpreted with caution. When compared with evidence from other surgical populations, the magnitude and pattern of magnesium’s analgesic effects in cardiothoracic surgery appear broadly similar but not superior. Prior meta-analyses in abdominal, orthopedic, and mixed surgical cohorts have generally reported modest reductions in early postoperative pain scores and opioid requirements, particularly when magnesium is administered as a continuous infusion or repeated dosing regimen, with attenuated or absent effects in low-dose or single-bolus protocols [30, 31]. Similar to findings in non-cardiac surgeries, the present review suggests that magnesium’s benefits are more evident when contrasted with placebo or standard care than when compared against potent active adjuvants, reinforcing its role as an additive component of multimodal analgesia rather than as a standalone or dominant agent [32,33]. The cardiothoracic-specific data extend this literature by confirming that magnesium can be used safely in a high-risk cardiac population, without clear hemodynamic or arrhythmic penalties, while delivering analgesic effects of comparable magnitude to those seen in other operative settings.​

Despite these encouraging observations, important evidence gaps remain. First, the overall certainty of evidence is limited by small sample sizes, variability in dosing strategies, and sparse data for several clinically relevant outcomes such as long-term pain, functional recovery, and quality of life. Second, optimal magnesium regimens covering route, loading and maintenance doses, timing relative to surgery, and duration of administrationhave not been systematically defined, particularly for specific cardiothoracic procedures such as off-pump CABG or minimally invasive valve surgery. Third, the comparative effectiveness of magnesium versus other commonly used adjuvants (e.g., dexmedetomidine, ketamine, gabapentinoids) in cardiothoracic patients is largely unknown, as few head-to-head trials exist. Finally, robust safety data on rare but clinically important adverse events, including serious arrhythmias, significant hypotension, or clinically meaningful hypermagnesemia, are limited by the small number of events and short follow-up periods.​

This review has several strengths that enhance the robustness and applicability of its findings, alongside limitations that warrant caution in interpretation. Strengths include a comprehensive search strategy across major databases and trial registries, prospective PROSPERO registration, adherence to PRISMA guidelines, restriction to randomized controlled trials, standardized opioid conversion to morphine milligram equivalents, and duplicate processes for study selection, data extraction, and risk-of-bias assessment using the RoB-2 tool. Focusing exclusively on cardiothoracic surgery yields clinically coherent insights for a high-risk population commonly experiencing moderate to severe postoperative pain. However, limitations include substantial statistical heterogeneity across most pooled outcomes, heterogeneity in surgical procedures and perioperative care, variation in magnesium dosing regimens and routes, and non-uniform outcome definitions and time points, all of which reduce the precision and generalizability of pooled estimates. The modest number of included trials and small sample sizes per outcome limit power to detect rare adverse events and preclude formal evaluation of publication bias, further lowering confidence in the certainty of effect.​

## Conclusion

Perioperative magnesium may offer modest improvements in postoperative pain after cardiothoracic surgery and appears to have an acceptable safety profile. However, the clinical relevance of these findings is uncertain given the substantial heterogeneity, limited number of studies, and variability in dosing regimens. Magnesium may be considered as a potential adjunct within multimodal analgesia strategies, but further well-designed trials are needed to clarify its effectiveness, optimal dosing, and appropriate clinical use.

## Supplementary Information

Supplementary Material 1.

Supplementary Material 2.

## Data Availability

No datasets were generated or analysed during the current study.

## Abbrevations

CABG: Coronary artery bypass grafting
CENTRAL: Cochrane central register of controlled trials
CI: Confidence interval
CRD: Centre for reviews and dissemination
EMBASE: Excerpta medica database
ICU: Intensive care unit
I²: I-squared statistic
MCID: Minimal clinically important difference
MD: Mean difference
Mg: Magnesium
MME: Morphine milligram equivalents
NMDA: N-methyl-D-aspartate
NSAIDs: N-methyl-D-aspartate
PRISMA: Preferred reporting items for systematic reviews and meta-analyses
PROSPERO: International prospective register of systematic reviews
RCT: Randomized controlled trial
REML: Restricted maximum likelihood
RoB-2: Risk of bias tool version 2
RR: Risk ratio
VAS: Visual analogue scale
VATS: Video-assisted thoracoscopic surgery
