# Supplementary material for: Efficacy and safety of perioperative magnesium on postoperative pain in adult patients undergoing cardiothoracic surgeries- a systematic review and meta-analysis
Source: J Cardiothorac Surg. 2026 May 7;21:462. doi: 10.1186/s13019-026-04252-0 (PMC13321671; doi:10.1186/s13019-026-04252-0)
Supplement: Supplementary file 1 — Supplementary Material 1. [file 13019_2026_4252_MOESM1_ESM.docx]

**SUPPLEMENTARY FILES**

**Supplementary table 1 :** Protocol deviation

| **Domain** | **Original Protocol Specification** | **Amendment / Deviation** | **Rationale / Timing** |
| --- | --- | --- | --- |
| **Primary outcome timepoint** | Primary pain outcome specified but without defined timepoint. | Predefined VAS at 24 hours as the primary timepoint; 48 hours designated as a key secondary timepoint. | Decision made a priori before quantitative synthesis based on clinical relevance and completeness of reporting. |
| **Route of magnesium administration** | Route not explicitly specified. | Included both intravenous and epidural administration routes. | Broadened scope to evaluate overall perioperative analgesic effects rather than route-specific pharmacokinetics; decision made a priori during screening, prior to data extraction. |
| **Secondary outcomes** | Did not include length of stay. | Added “length of stay” as a secondary outcome. | To capture broader recovery-related outcomes and postoperative recovery metrics. |
| **Population (age restriction)** | No age restriction specified. | Restricted to adult patients (≥18 years). | To enhance population homogeneity and reduce variability in analgesic response. |
| **Statistical software** | STATA 18 SE specified for analysis. | Used R Studio (version 4.3.2) with the metafor package for data synthesis. | R offered more advanced meta-analysis and visualization tools; statistical methods remained consistent. |

**Supplementary Table 2:** Search strategy in Pubmed,Scopus,Cochrane, Embase

| **Pubmed** | | |
| --- | --- | --- |
| Framework | Search Terms | Search Hits as on 14/05/2025 |
| P1-Population (Patients undergoing cardiothoracic surgeries) | cardio OR cardiac OR "heart"[MeSH Terms] OR heart OR CABG OR "Coronary Artery Bypass"[Mesh] OR coronary OR "valve replacement" OR thoracal OR thoracical OR "thorax"[MeSH Terms] OR thorax OR thoracic OR thoracics OR "Lung Resection" OR Esophagectomy OR "Esophagectomy"[Mesh] OR thoracotomy OR "Thoracotomy"[Mesh] OR lobectomy OR pneumonectomy OR "Pneumonectomy"[Mesh] | [2,909,590](https://pubmed.ncbi.nlm.nih.gov/?term=cardio+OR+cardiac+OR+%22heart%22%5BMeSH+Terms%5D+OR+heart+OR+CABG+OR+%22Coronary+Artery+Bypass%22%5BMesh%5D+OR+coronary+OR+%22valve+replacement%22+OR+thoracal+OR+thoracical+OR+%22thorax%22%5BMeSH+Terms%5D+OR+thorax+OR+thoracic+OR+thoracics+OR+%22Lung+Resection%22+OR+Esophagectomy+OR+%22Esophagectomy%22%5BMesh%5D+OR+thoracotomy+OR+%22Thoracotomy%22%5BMesh%5D+OR+lobectomy+OR+pneumonectomy+OR+%22Pneumonectomy%22%5BMesh%5D&sort=date&ac=no) |
| P2 | "Surgical Procedures, Operative"[Mesh] OR surgery OR surgical OR surgeries OR operative OR operation OR transplant* | [7,456,062](https://pubmed.ncbi.nlm.nih.gov/?term=%22Surgical+Procedures%2C+Operative%22%5BMesh%5D+OR+surgery+OR+surgical+OR+surgeries+OR+operative+OR+operation+OR+transplant%2A&sort=date&ac=no) |
| I-Intervention (Magnesium) | "magnesium"[Supplementary Concept] OR "Magnesium"[Mesh] OR magnesium | [126,406](https://pubmed.ncbi.nlm.nih.gov/?term=%22magnesium%22%5BSupplementary+Concept%5D+OR+%22Magnesium%22%5BMesh%5D+OR+magnesium&sort=date&ac=no) |
| O-Outcome | "pain"[MeSH Terms] OR pain* | [1,225,603](https://pubmed.ncbi.nlm.nih.gov/?term=%22pain%22%5BMeSH+Terms%5D+OR+pain%2A&sort=date&ac=no) |
| P AND I AND C AND O |  | [181](https://pubmed.ncbi.nlm.nih.gov/?term=%28%28%28cardio+OR+cardiac+OR+%22heart%22%5BMeSH+Terms%5D+OR+heart+OR+CABG+OR+%22Coronary+Artery+Bypass%22%5BMesh%5D+OR+coronary+OR+%22valve+replacement%22+OR+thoracal+OR+thoracical+OR+%22thorax%22%5BMeSH+Terms%5D+OR+thorax+OR+thoracic+OR+thoracics+OR+%22Lung+Resection%22+OR+Esophagectomy+OR+%22Esophagectomy%22%5BMesh%5D+OR+thoracotomy+OR+%22Thoracotomy%22%5BMesh%5D+OR+lobectomy+OR+pneumonectomy+OR+%22Pneumonectomy%22%5BMesh%5D%29+AND+%28%22Surgical+Procedures%2C+Operative%22%5BMesh%5D+OR+surgery+OR+surgical+OR+surgeries+OR+operative+OR+operation+OR+transplant%2A%29%29+AND+%28%22magnesium%22%5BSupplementary+Concept%5D+OR+%22Magnesium%22%5BMesh%5D+OR+magnesium%29%29+AND+%28%22pain%22%5BMeSH+Terms%5D+OR+pain%2A%29&sort=date&ac=no) |
| **Scopus** | | |
| Framework | Search Terms | Search Hits as on 14/05/2025 |
| P-Population | TITLE-ABS-KEY ( cardio OR cardiac OR heart OR cabg OR coronary OR "valve replacement" OR thoracal OR thoracical OR thorax OR thoracic OR thoracics OR "Lung Resection" OR esophagectomy OR thoracotomy OR lobectomy OR pneumonectomy ) | [3,906,207 results](https://www.scopus.com/results/results.uri?s=TITLE-ABS-KEY%28cardio+OR+cardiac+OR+heart+OR+CABG+OR+coronary+OR+%22valve+replacement%22+OR+thoracal+OR+thoracical+OR+thorax+OR+thoracic+OR+thoracics+OR+%22Lung+Resection%22+OR+Esophagectomy+OR+thoracotomy+OR+lobectomy+OR+pneumonectomy%29&limit=10&origin=searchhistory&sort=plf-f&src=s&sot=b&sdt=b&sessionSearchId=91be65315523370aa0f19865973a41bc) |
| P2 | TITLE-ABS-KEY ( surgery OR surgical OR surgeries OR operative OR operation OR transplant* ) | [7,754,543 results](https://www.scopus.com/results/results.uri?s=TITLE-ABS-KEY%28surgery+OR+surgical+OR+surgeries+OR+operative+OR+operation+OR+transplant*%29&limit=10&origin=searchhistory&sort=plf-f&src=s&sot=b&sdt=b&sessionSearchId=9202d282c12758054c1ccee10cb14847) |
| I- Intervention | TITLE-ABS-KEY ( magnesium ) | [456,973 results](https://www.scopus.com/results/results.uri?s=TITLE-ABS-KEY%28magnesium%29&limit=10&origin=searchhistory&sort=plf-f&src=s&sot=b&sdt=b&sessionSearchId=f3e35649e1e8e146fae65f40113e08db) |
| O-Outcome | ALL ( pain* ) | [4,217,898 results](https://www.scopus.com/results/results.uri?s=ALL%28pain*%29&limit=10&origin=searchhistory&sort=plf-f&src=s&sot=b&sdt=b&sessionSearchId=dcf348874a1646d2639dc0a1cb5f1368) |
| P AND I AND C AND O |  | [1,090 results](https://www.scopus.com/results/results.uri?s=%28TITLE-ABS-KEY+%28+cardio+OR+cardiac+OR+heart+OR+cabg+OR+coronary+OR+%22valve+replacement%22+OR+thoracal+OR+thoracical+OR+thorax+OR+thoracic+OR+thoracics+OR+%22Lung+Resection%22+OR+esophagectomy+OR+thoracotomy+OR+lobectomy+OR+pneumonectomy+%29%29+AND+%28TITLE-ABS-KEY+%28+surgery+OR+surgical+OR+surgeries+OR+operative+OR+operation+OR+transplant*+%29%29+AND+%28TITLE-ABS-KEY+%28+magnesium+%29%29+AND+%28ALL+%28+pain*+%29%29&limit=10&origin=searchhistory&sort=plf-f&src=s&sot=comb&sdt=comb&sessionSearchId=b29c08824f51fe17263088a679499c01) |
| **Cochrane** | | |
| Framework | Search Terms | Search Hits as on 14/05/2025 |
| Framework | Search Terms | Search Hits as on 14/05/2025 |
| P-Population | cardio OR cardiac OR heart OR CABG OR coronary OR "valve replacement" OR thoracal OR thoracical OR thorax OR thoracic OR thoracics OR "Lung Resection" OR Esophagectomy OR thoracotomy OR lobectomy OR pneumonectomy |  |
| P2 | surgery OR surgical OR surgeries OR operative OR operation OR transplant* |  |
| I-Intervention | magnesium |  |
| O-Outcome | pain* |  |
| P AND I AND C AND O |  | 333 |
| **Embase** | | |
| Framework | Search Terms | Search Hits as on 14/05/2025 |
| P-Population | cardio:ti,ab,kw OR cardiac:ti,ab,kw OR heart:ti,ab,kw OR CABG:ti,ab,kw OR coronary:ti,ab,kw OR ‘valve replacement’:ti,ab,kw OR thoraca:ti,ab,kwl OR thoracical:ti,ab,kw OR thorax:ti,ab,kw OR thoracic:ti,ab,kw OR thoracics:ti,ab,kw OR ‘Lung Resection’:ti,ab,kw OR Esophagectomy:ti,ab,kw OR thoracotomy:ti,ab,kw OR lobectomy:ti,ab,kw OR pneumonectomy:ti,ab,kw | 2,970,493 |
| P2 | surgery:ti,ab,kw OR surgical:ti,ab,kw OR surgeries:ti,ab,kw OR operative:ti,ab,kw OR operation:ti,ab,kw OR transplant:ti,ab,kw | 4,245,279 |
| I- Intervention | magnesium:ti,ab,kw | 93,819 |
| O-Outcome | pain* | 2,385,240 |
| P AND I AND C AND O |  | 293 |

**Supplementary table 3**: Risk of bias assessment (RoB 2.0): D1–D5

represent bias domains; (+) low risk, (–) some concerns, (X) high risk.


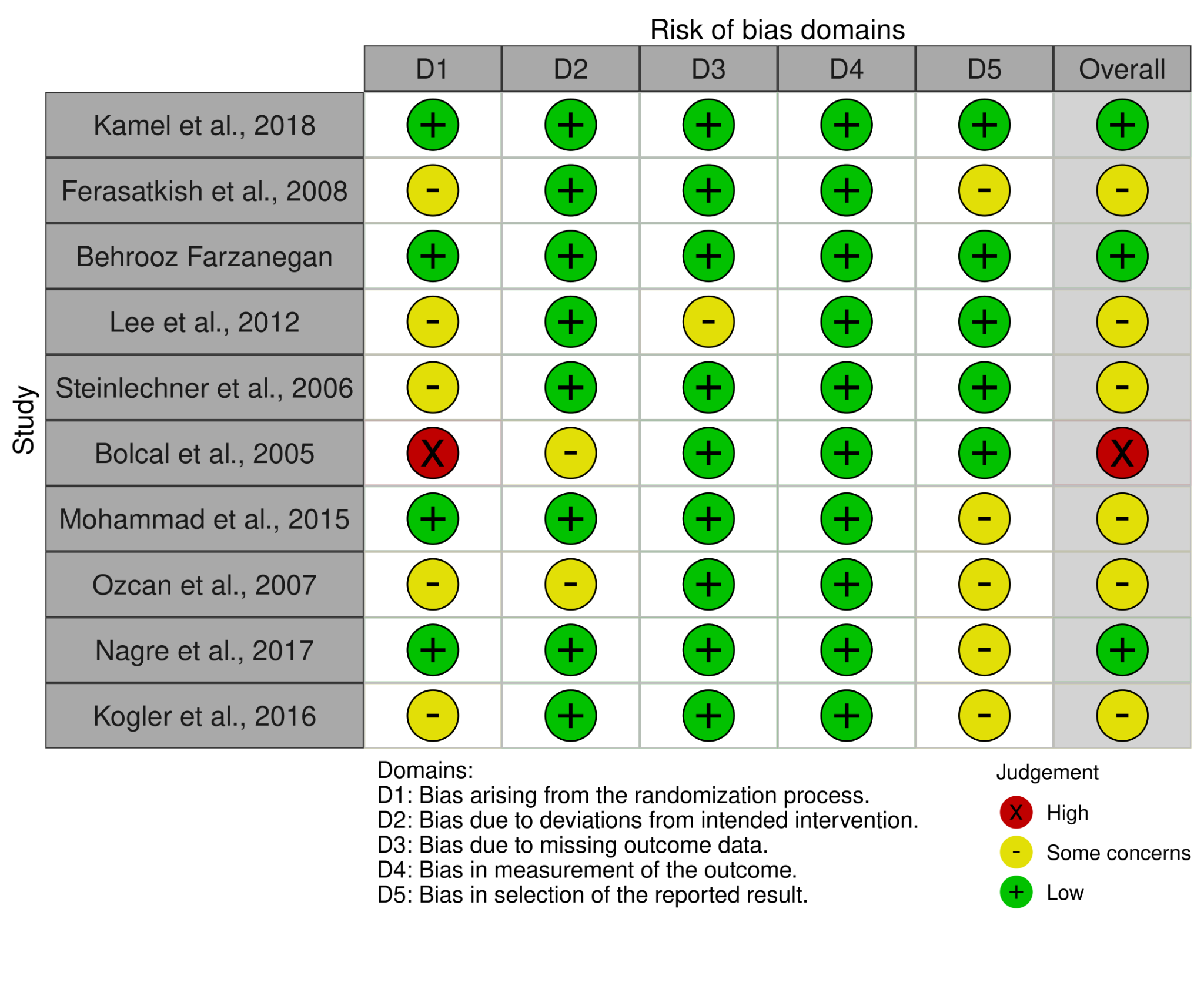


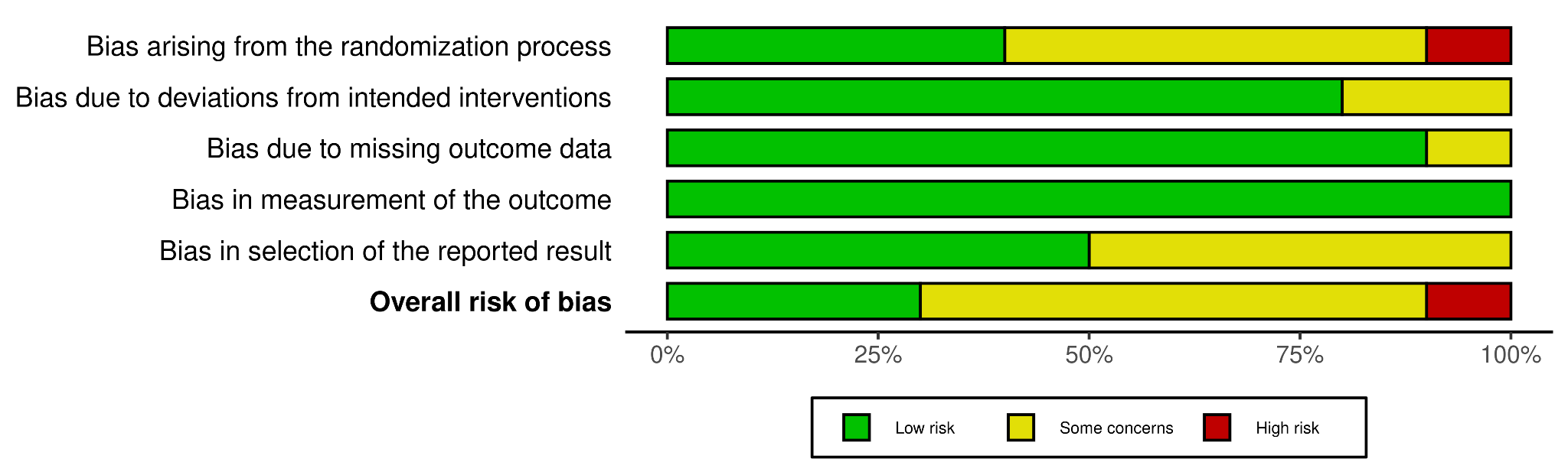


**Supplementary table 4:** List of excluded studies

| **S.No** | **List of Excluded studies** | **Reason** |
| --- | --- | --- |
| **1** | **Effect of intraoperative magnesium on the incidence of chronic post surgical pain after thoracotomy** | **Wrong Outcome** |
| **2** | **Dexmedetomidine versus magnesium in thoracoscop** | **Wrong Outcome** |
| **3** | **Effect of intraoperative systemic magnesium sulphate on postoperative Richmond Agitation-Sedation Scale score after endovascular repair of aortic aneurysm under general anesthesia: A double-blind, randomized, controlled trial** | **Wrong Score** |
| **4** | **Postoperative pain management in adult cardiac surgery** | **Textbook** |

| 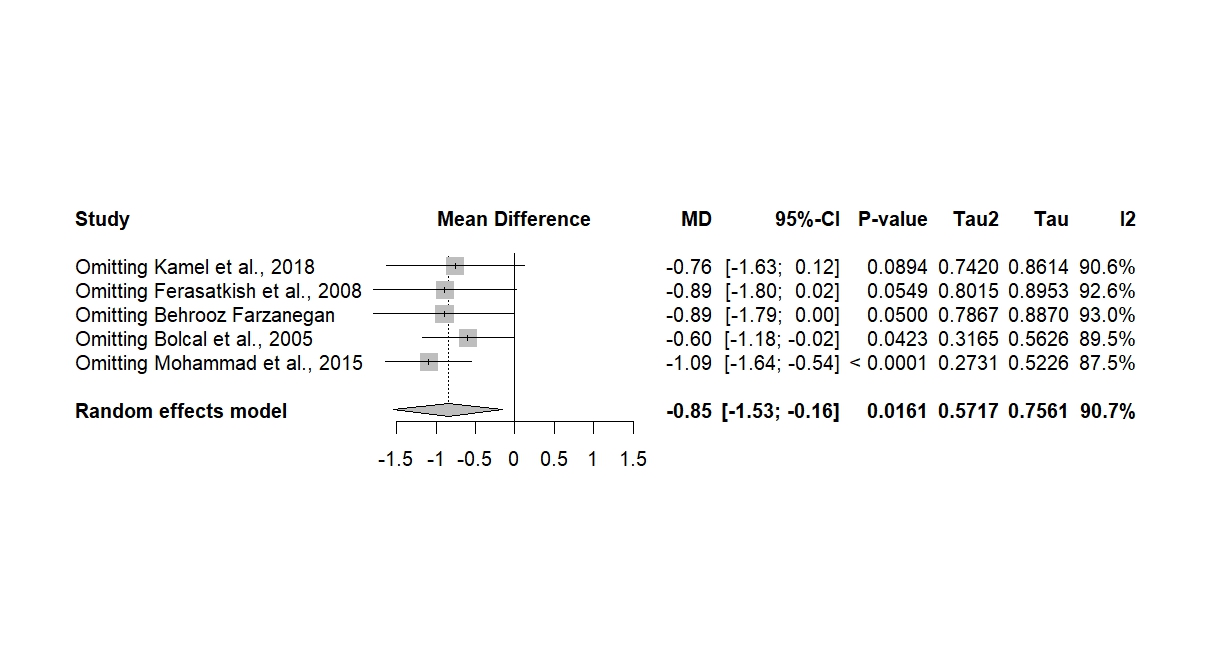 |
| --- |
| **Supplementary Figure 1**:Sensitivity analysis (leave-one-out method) comparing pain scores at 24 hours between the magnesium group and the normal saline group. |

| 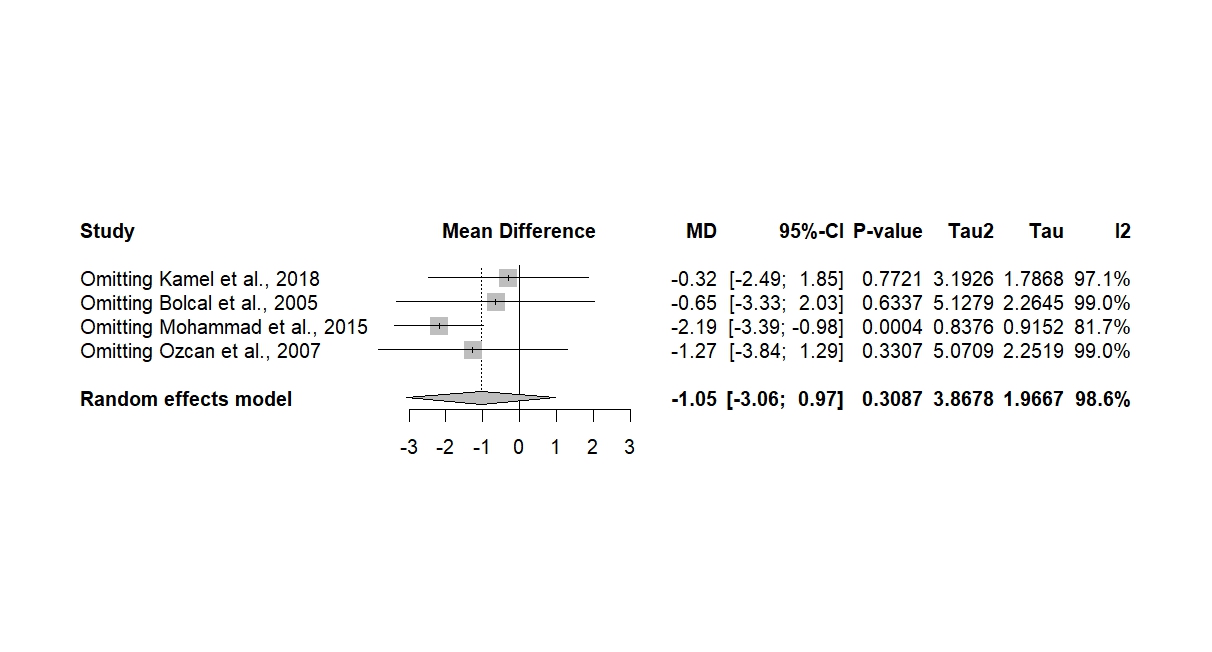 |
| --- |
| **Supplementary Figure 2:** Sensitivity analysis (leave-one-out method) comparing pain scores at 24 hours between the magnesium group and the analgesia group. |

| 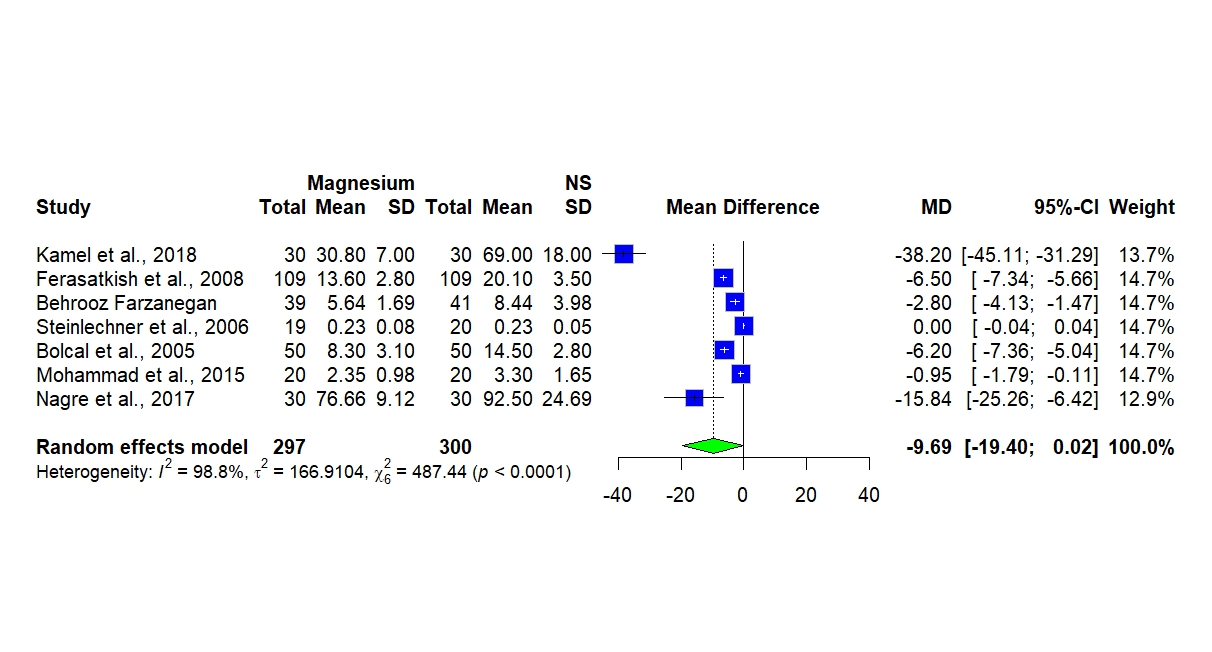 |
| --- |
| **Supplementary Figure 3:**Forest plot showing pooled mean difference in Analgesic consumption between magnesium and saline groups |

| 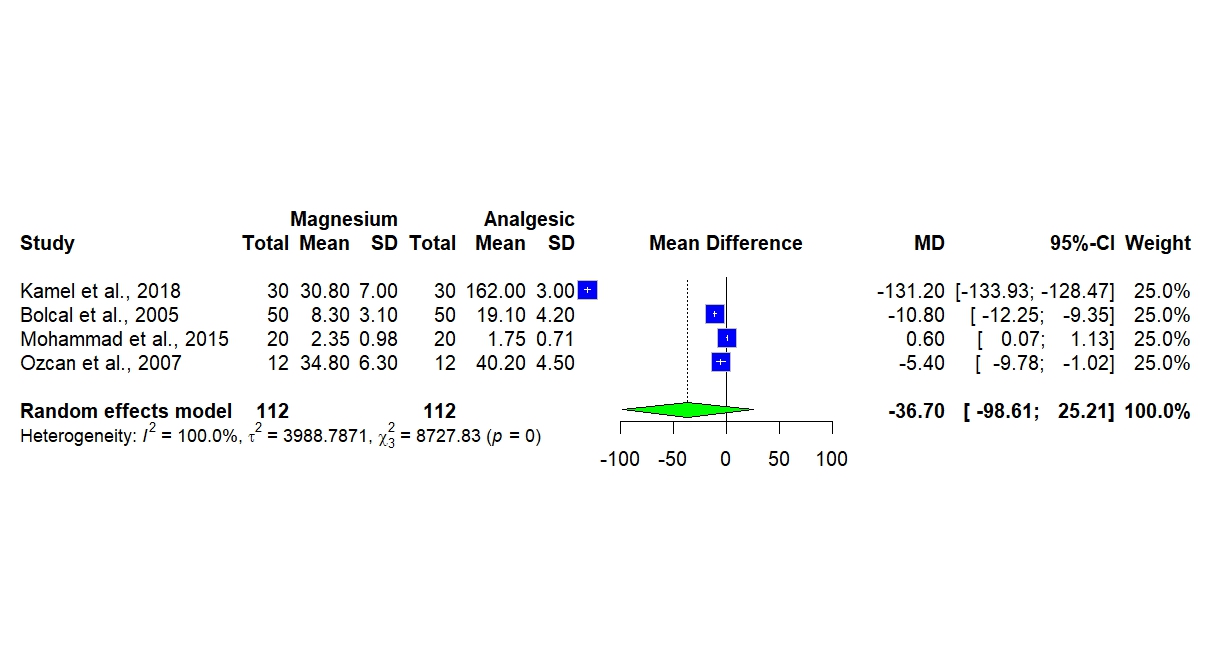 |
| --- |
| **Supplementary Figure 4**:Forest plot showing pooled mean difference in analgesic consumption between magnesium and concomitant analgesic group |

| 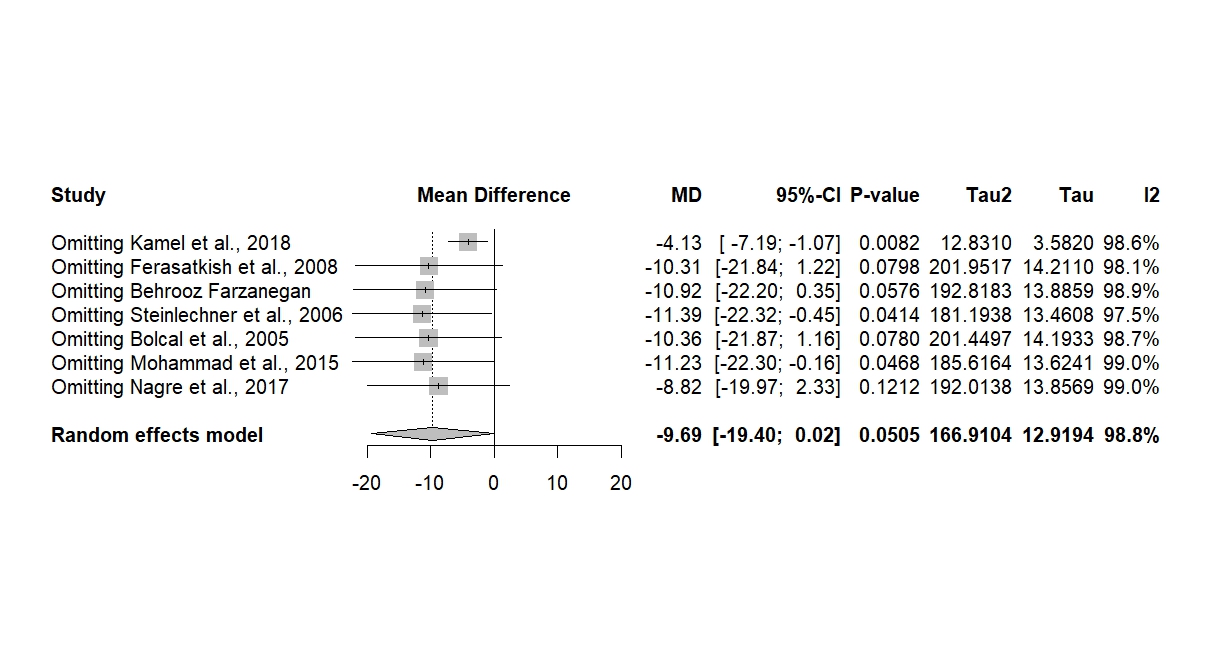 |
| --- |
| **Supplementary Figure 5:**.Sensitivity analysis (leave-one-out method) comparing pain scores at 24 hours between the magnesium group and the normal saline group |

| 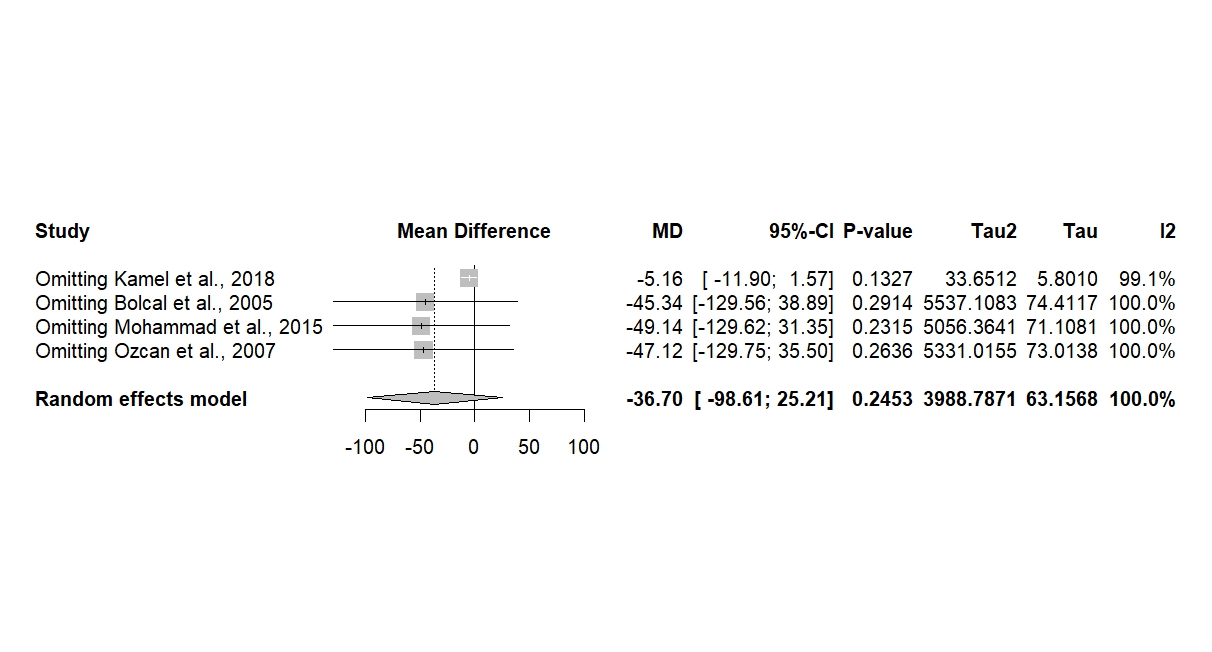 |
| --- |
| **Supplementary Figure 6**: .Sensitivity analysis (leave-one-out method) comparing pain scores at 24 hours between the magnesium group and the normal saline group |

| 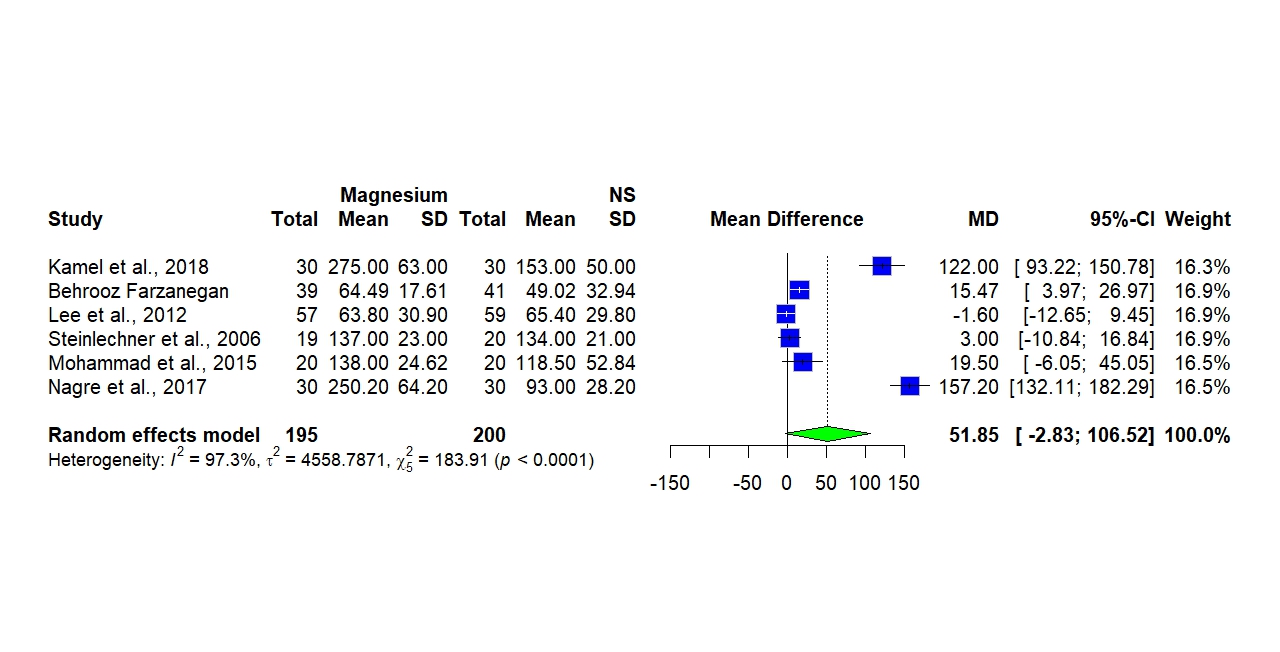 |
| --- |
| **Supplementary Figure 7:** Forest plot showing pooled mean difference in time to first rescue analgesia between magnesium and normal saline group |

| 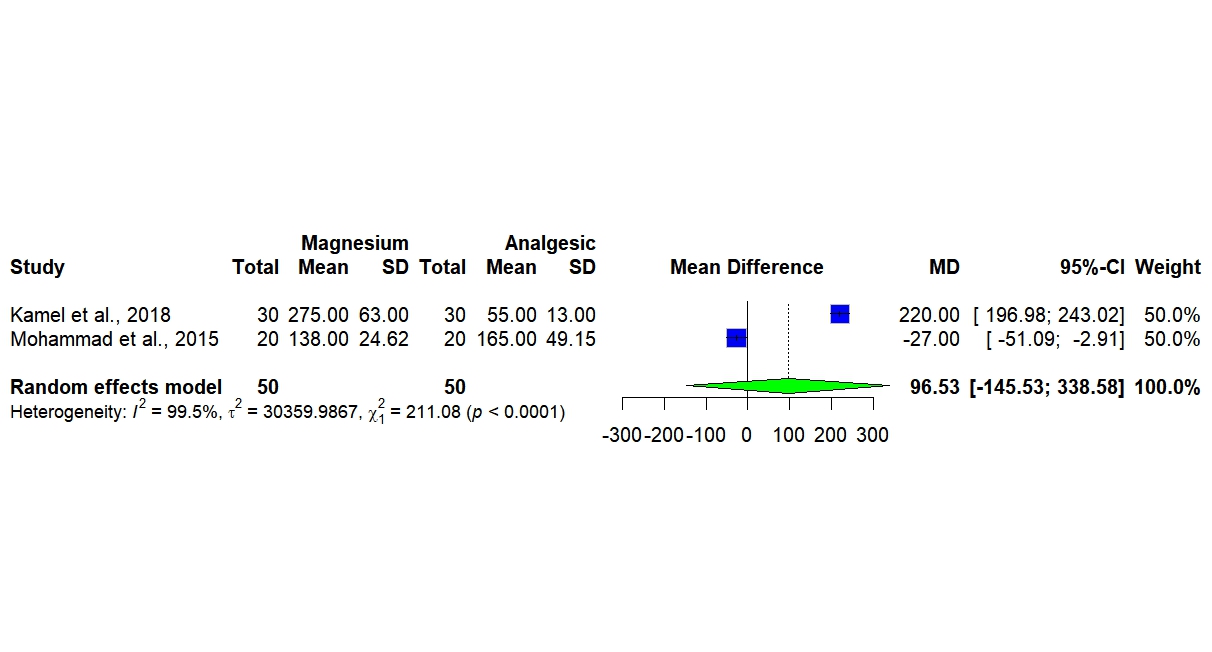 |
| --- |
| **Supplementary Figure 8**:Forest plot showing pooled mean difference in time to first rescue analgesia between magnesium and analgesic group |

| 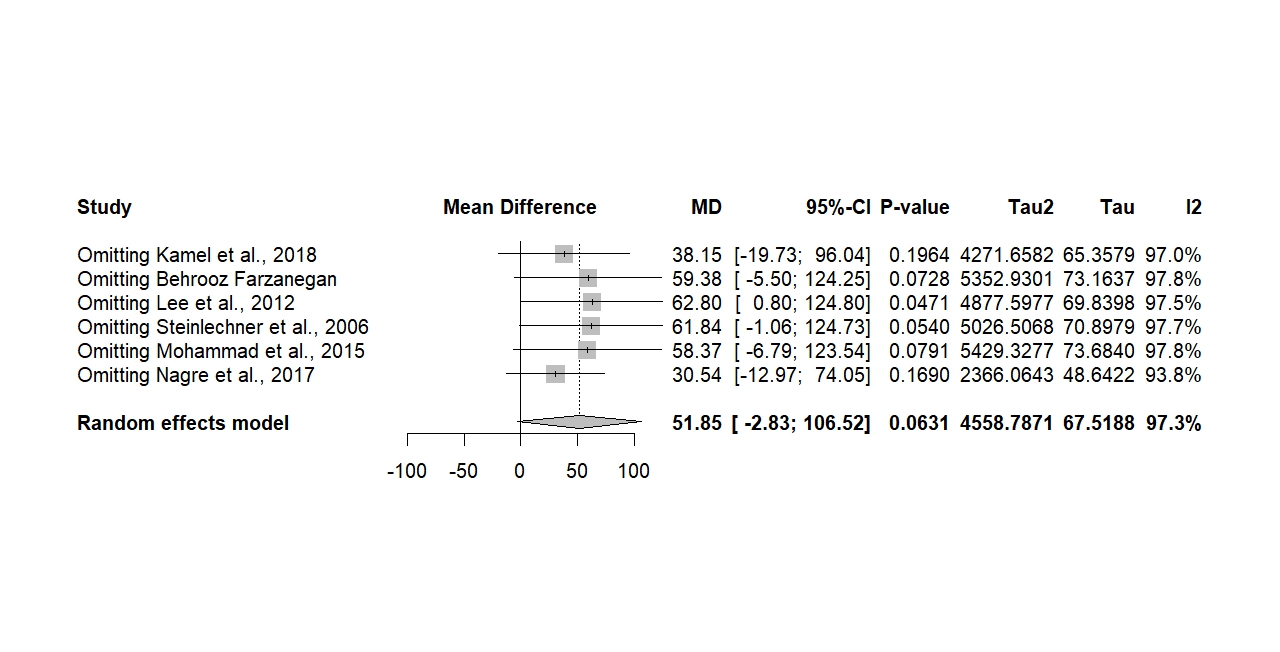 |
| --- |
| **Supplementary Figure 9:** Sensitivity analysis (leave-one-out method) comparing pain scores at 24 hours between the magnesium group and the normal saline group |

| 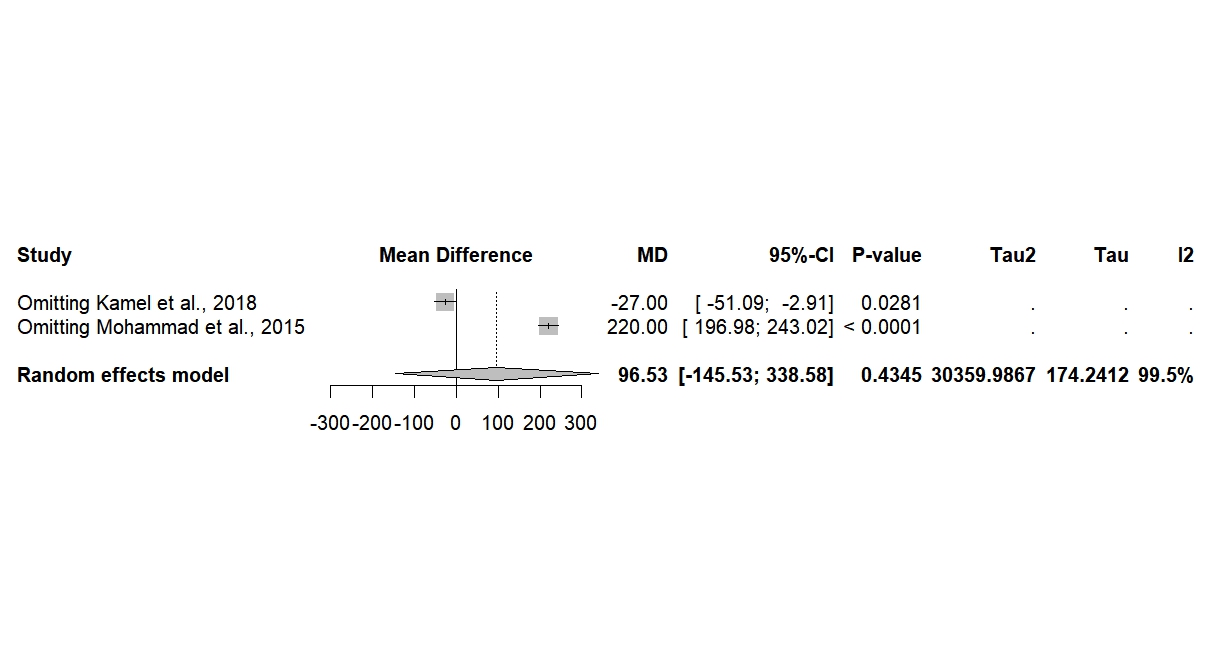 |
| --- |
| **Supplementary Figure 10:**Sensitivity analysis (leave-one-out method) comparing pain scores at 24 hours between the magnesium group and the normal saline group |

| 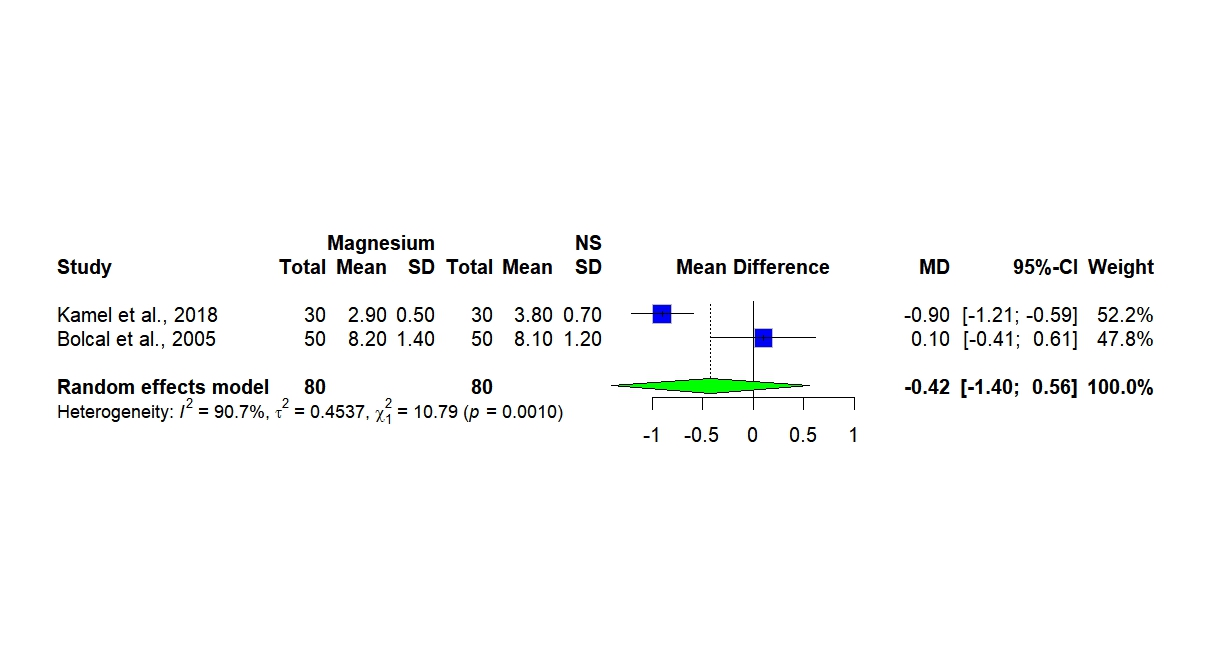 |
| --- |
| **Supplementary Figure 11:** Forest plot showing pooled mean difference in length of stay between magnesium and normal saline. |

| 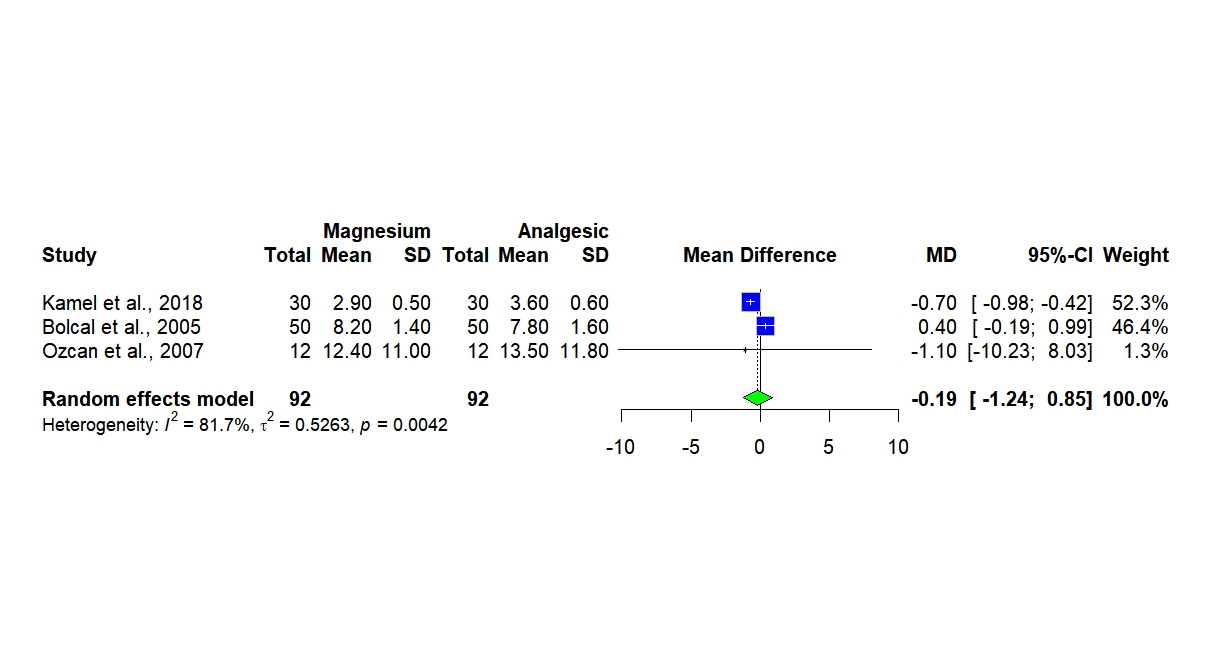 |
| --- |
| **Supplementary Figure 12** : Forest plot showing pooled mean difference in length of stay between magnesium and analgesic group |

| 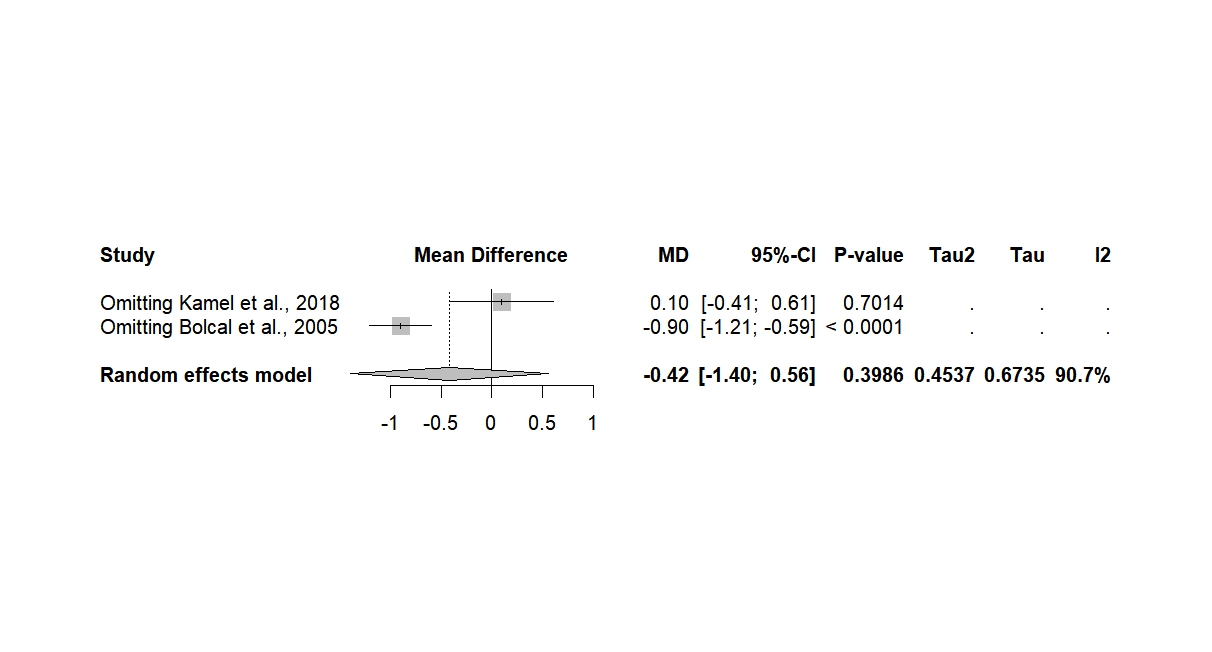 |
| --- |
| **Supplementary Figure 13:**Sensitivity analysis (leave-one-out method) comparing pain scores at 24 hours between the magnesium group and the normal saline group |

| 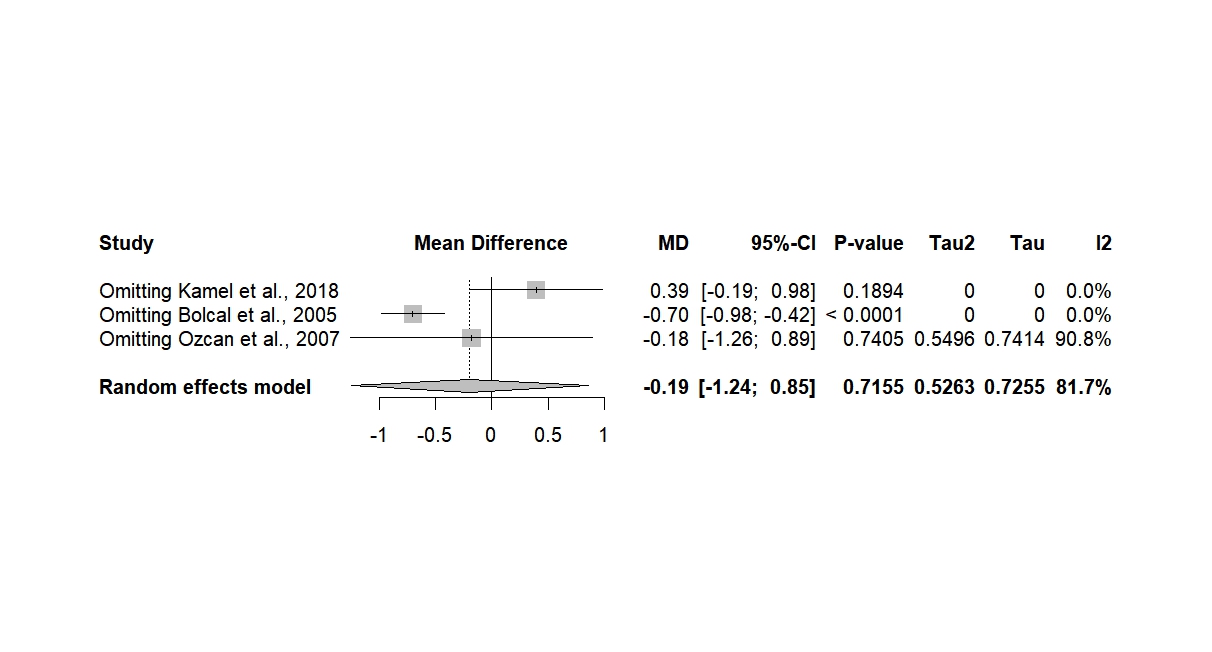 |
| --- |
| **Supplementary Figure 14:** Sensitivity analysis (leave-one-out method) comparing pain scores at 24 hours between the magnesium group and the normal saline group |
